# Research mentoring and scientist identity: insights from undergraduates and their mentors

**DOI:** 10.1186/s40594-018-0139-y

**Published:** 2018-11-30

**Authors:** Rachael D Robnett, Paul A Nelson, Eileen L Zurbriggen, Faye J Crosby, Martin M Chemers

**Affiliations:** 1 Department of Psychology, University of Nevada, Las Vegas, Las Vegas, NV USA; 2 Department of Psychology, University of California, Santa Cruz, Santa Cruz, CA USA

**Keywords:** Mentoring, Science Identity, Diversity, Post-secondary

## Abstract

**Background:**

Mentored research apprenticeships are a common feature of academic outreach programs that aim to promote diversity in science fields. The current study tests for links between three forms of mentoring (instrumental, socioemotional, and negative) and the degree to which undergraduates psychologically identify with science. Participants were 66 undergraduate-mentor dyads who worked together in a research apprenticeship. The undergraduate sample was predominantly composed of women, first-generation college students, and members of ethnic groups that are historically underrepresented in science.

**Results:**

Findings illustrated that undergraduates who reported receiving more instrumental and socioemotional mentoring were higher in scientist identity. Further, mentors who reported engaging in higher levels of negative mentoring had undergraduates with lower scientist identity. Qualitative data from undergraduates’ mentors provided deeper insight into their motivation to become mentors and how they reason about conflict in their mentoring relationships.

**Conclusions:**

Discussion highlights theoretical implications and details several methodological recommendations.

Mentoring relationships occur when someone more experienced (i.e., the mentor) takes responsibility for guiding and supporting someone less experienced (i.e., the protégé). It is arguably a truism to state that mentoring relationships can have important implications for protégés. Literature reviews and meta-analyses have linked mentoring to a variety of protégé outcomes that range from self-reported wellbeing to objective performance metrics (Eby et al. 2013; Jacobi 1991). Many of these findings originate from workplace and organizational settings, where mentoring relationships have long been the focus of empirical attention (e.g., Kram 1985). More recently, mentoring has been identified as a promising strategy for addressing the dearth of diversity in fields related to science (Haeger and Fresquez 2016; Lopatto 2007; Syed and Chemers 2011), which is a serious economic concern within the USA (e.g., President’s Council of Advisors on Science and Technology 2012). Specifically, undergraduates—particularly those who are members of underrepresented groups—appear to benefit in many ways from contributing to scientific research under the guidance of more experienced researchers (Hernandez et al. 2018; Russell et al. 2007; Sadler et al. 2010).

The current study examines whether mentoring experiences in science research apprenticeships predict the extent to which undergraduates psychologically identify with science. We examined this question in a sample of undergraduates who are members of groups that are traditionally underrepresented in science. Our methodological approach differs from prior research in two main ways. First, research typically relies on accounts from students to understand mentoring dynamics. In contrast, the current study utilizes data from student-mentor dyads. Second, we expand on research that has mainly focused on the positive aspects of mentoring relationships by considering negative aspects as well. Below, we further describe scientist identity and its hypothesized association with research mentoring.

## Scientist identity in students from underrepresented groups

We define *scientist identity* as the degree to which students perceive their science-related pursuits as integral to their sense of self. By this definition, students with a strong scientist identity perceive “scientist” as an important component of their self-image and derive personal meaning from their scientific endeavors. Scientist identity is associated with positive academic outcomes such as a heightened likelihood of persistence in the science pipeline (Aschbacher et al. 2010; Estrada et al. 2011; Chemers et al. 2011; Hazari et al. 2010; Syed et al. in press). For instance, Hazari et al. (2010) demonstrated that students who were higher in physics identity were much more likely than other students to anticipate having a career in physics. Similarly, Chemers et al. (2011) found strong associations between scientist identity and commitment to a science career among undergraduates, graduate students, and postdoctoral students in science fields.

Forging a strong scientist identity may present unique challenges for members of groups that are historically underrepresented in science, such as women, first-generation college students, and members of some ethnic minority groups (Syed 2010; Syed et al. 2011). For example, some members of underrepresented ethnic groups may avoid or leave science-related fields because they perceive the contextual norms in these fields as incompatible with aspects of their ethnic or gender identity (Cheryan et al. 2009; Syed 2010). Another concern is that members of underrepresented groups receive subtle and overt messages that they do not belong in science and academia more generally (Lohfink and Paulsen 2005; Ong et al. 2011; Robnett 2016). For instance, Carlone and Johnson (2007) found that some Women of Color in science were characterized by “disrupted” scientist identities. The disruption was caused by people in positions of power (e.g., professors, research supervisors) focusing on the women’s sociodemographic background instead of recognizing them as skilled scientists. Similarly, Robnett (2016) found that the majority of women in science majors experienced sexism, which often took the form of others expecting less of them because of their gender. These findings illustrate the importance of identifying strategies that can instill and reinforce scientist identity among students from underrepresented groups.

## Science research mentoring

In the current study, we examine whether research mentoring has implications for undergraduates’ scientist identity. Research mentoring occurs when students participate in scientific research under the guidance of more experienced students and faculty. Involvement in authentic, hands-on research has several advantages over lecture-based models of science education (Chin and Malhotra 2002; Handelsman et al. 2004; Sadler et al. 2010). Specifically, research involvement helps integrate students into science communities of practice (see Lave and Wenger 1991), wherein they obtain firsthand insight into the norms, behaviors, and ways of thinking that characterize successful scientists (Aschbacher et al. 2010; Hunter et al. 2007). Membership in these communities of practice can enhance belongingness, which is an integral component of identity (Carlone and Johnson 2007; Cohen and Garcia 2008; Syed et al. 2011). Put differently, students who feel like they belong in science are more likely than other students to develop a strong scientist identity.

### Insights from students and their mentors

When examining the impact of science research apprenticeships, prior research tends to focus on how students characterize the mentoring relationship (e.g., Chemers et al. 2011; Tenenbaum et al. 2001). However, there may be advantages to simultaneously considering data from students *and* their mentors. When evaluating the mentoring relationship, mentors have the advantage of reflecting on all of their past mentoring experiences (see Hunter et al. 2007). It is therefore plausible that, relative to students, mentors provide more refined or realistic evaluations of the mentoring relationship. Thus, although students’ appraisals of the mentoring they receive are valuable from a predictive standpoint (e.g., Tenenbaum et al. 2001), we expected that mentors’ self-ratings might explain additional variance in student outcomes. In the few studies that do collect data from both parties, mentors often provide information that helps to contextualize or clarify student accounts (Hunter et al. 2007; Kardash 2000; Kardash and Edwards 2012). For instance, Kardash and Edwards (2012) found that students and their mentors sometimes differed in their beliefs about the thought patterns and behaviors that characterize scientists.

The current study continues along this vein by examining data collected from student-mentor dyads. More specifically, students and their research mentors both reported on the prevalence of various mentoring behaviors, and we examined whether their reports independently predicted students’ scientist identity. Our interest in examining mentors’ ratings of their own mentoring behaviors was grounded in the previously described work indicating that mentors serve as a gateway into science communities of practice. Accordingly, the mentoring relationship is likely to be an important context for the development of scientist identity (e.g., Carlone and Johnson 2007). To our knowledge, prior research has not compared students’ evaluations of the mentoring they receive to their research mentors’ evaluations of the mentoring they provide. However, within organizational settings, evidence suggests that protégé and mentor ratings of mentoring work in tandem to predict protégé outcomes (Godshalk and Sosik 2000).

### Instrumental, socioemotional, and negative mentoring

The mentoring that students receive in their research apprenticeships can take several forms. Two widely discussed forms of mentoring are instrumental mentoring and socioemotional mentoring (Eby et al. 2013; Kram 1985; Tenenbaum et al. 2001). *Instrumental mentoring* is task-focused mentoring; it involves providing the protégé with skills and resources that help the protégé succeed in a given context. For instance, a research mentor is engaged in instrumental mentoring when she helps her student learn a new research method or statistical technique. In contrast, *socioemotional mentoring* involves providing the protégé with social and emotional support. For instance, a research mentor is engaged in socioemotional mentoring when she gives her student a motivational talk before an important presentation. In line with prior research (e.g., Chemers et al. 2011), we anticipated that higher levels of instrumental and socioemotional mentoring would be associated with higher scientist identity for students in the current study.

Instrumental and socioemotional mentoring are typically conceptualized as positive mentoring behaviors. As with most meaningful relationships, however, mentoring relationships can also have difficult moments (see Wood and Duck 1995). *Negative mentoring* occurs when the mentor engages in practices that undermine the mentoring bond (Eby et al. 2010; Eby et al. 2000). For instance, a mentor who routinely cancels meetings with her student or makes demeaning remarks about her student’s work is engaged in negative mentoring.

Little is known about how negative mentoring manifests in science research apprenticeships. Indeed, the mentoring literature in general tends not to focus on the negative aspects of mentoring relationships, particularly when it comes to the mentor’s perceptions of negativity (for discussion, see Eby and Allen 2002; Scandura 1998). Several studies conducted in workplace settings suggest that negative mentoring merits greater empirical attention. For instance, mentoring relationships characterized by high levels of negativity can contribute to psychological distress and turnover intentions for protégés (Eby and Allen 2002; Eby et al. 2010).

The current study extends this work in two ways. First, we examined whether undergraduates who receive more negative mentoring tend to have lower scientist identity. Second, we sought to provide deeper insight into how mentors experience and reason about conflict in the mentoring relationship. Among mentors who perceive conflict, we were particularly interested in distinguishing between those who take joint responsibility for conflict versus blaming the student. Social exchange theory (Cook and Rice 2003) and corresponding research from organizational settings (e.g., DeChurch and Marks 2001) suggest that mentors who take a “we’re in this together” approach to managing conflict may be especially effective in promoting positive student outcomes such as scientist identity development.

## Present study

The theory and research reviewed thus far indicate that students’ experiences in science research apprenticeships may have implications for their scientist identity. The current study builds on this work by (1) examining data from undergraduates and their research mentors and (2) focusing on both positive and negative aspects of mentoring relationships. This approach aligns with recommendations from Eby et al. (2010), who emphasized the importance of considering “both good and bad mentoring experiences, and doing so from both the protégé and the mentor perspective” (p. 89).

Given the novelty of our research design, we advanced a combination of exploratory research questions and hypotheses. First, we examined whether undergraduates and their mentors are “on the same page” about the amount of mentoring that mentors provided. Specifically, our first research question is as follows:

RQ1: Do undergraduates and their research mentors significantly differ in their perceptions of the amount of instrumental, socioemotional, and negative mentoring provided by the mentor?

Next, we examined whether undergraduates’ scientist identity was associated with undergraduates’ and their mentors’ reports of instrumental, socioemotional, and negative mentoring. As noted earlier, mentor reports appear to contribute important information above and beyond student data (e.g., Kardash 2000). Accordingly, we anticipated that undergraduates’ and their mentors’ characterizations of the mentoring relationship would independently predict undergraduates’ scientist identity when simultaneously entered into a regression model. Specifically, we advanced the following predictions:

H1a: Higher levels of instrumental mentoring will be associated with higher scientist identity. That is, when undergraduates report receiving more instrumental mentoring, their scientist identity will be higher. Similarly, when mentors report providing more instrumental mentoring, their undergraduates’ scientist identity will be higher.

H1b: Higher levels of socioemotional mentoring will be associated with higher scientist identity. That is, when undergraduates report receiving more socioemotional mentoring, their scientist identity will be higher. Similarly, when mentors report providing more socioemotional mentoring, their undergraduates’ scientist identity will be higher.

H1c: Higher levels of negative mentoring will be associated with lower scientist identity. That is, when undergraduates report receiving more negative mentoring, their scientist identity will be lower. Similarly, when mentors report providing more negative mentoring, their undergraduates’ scientist identity will be lower.

Last, accounts from mentors are only rarely examined in prior research. Accordingly, we conducted exploratory analyses that focused on two sources of data from mentors. First, to provide deeper insight into the context of the mentoring relationship, we sought to identify motivating factors that lead mentors to engage in research mentoring. Second, we examined how mentors characterized difficulties within the student-mentor relationship. In the tradition of prior mentoring research (e.g., Eby et al. 2000), we utilized qualitative data to answer the following research questions:

RQ2: What motivating factors lead mentors to engage in research mentoring? To what extent is educational equity a salient motivation?

RQ3a: According to mentors, what are the most common sources of difficulty and conflict in the relationship with their students? Do the types of difficulties that mentors report vary depending on students’ scientist identity?

RQ3b: When conflict is present, how do mentors attribute blame? Does their tendency to shoulder blame vary depending on students’ scientist identity?

## Method: undergraduate sample

### Undergraduate participants

The current study utilizes survey data from undergraduates and their science research mentors. Undergraduates and their mentors were affiliated with a variety of 2-year and 4-year colleges and universities across the USA. We recruited undergraduate participants through a partnership with an educational outreach organization that promotes diversity in science fields.^1^ More specifically, undergraduates who registered to attend the organization’s national research conference received emails from the research team requesting their participation in the current study. Most of the undergraduate participants (74%) presented posters at the conference. The recruiting email described the study as “a project designed to help us learn about the ‘active ingredients’ that support science students most effectively.” It also explained that participants would receive a $50 gift card in exchange for their participation. The email concluded with a survey link that allowed participants to access the consent form and online survey. Approximately 200 undergraduates were recruited, and 121 (61%) completed the survey. Of the 121 survey respondents, 66 undergraduates (55%) met the criteria for the current study. Specifically, they (a) reported having a mentor, (b) granted us permission to contact their mentor, and (c) had a mentor who responded to the survey. These 66 undergraduate-mentor dyads are the focus of the current study.

Of the 66 undergraduate participants, the mean age was 24.58 years (SD = 6.66, range = 18–51). Most participants were juniors (29%) or seniors (58%); however, first-years (1%) and sophomores (12%) also participated. All participants reported that they were majoring in fields related to science. Consistent with the outreach organization’s mission of diversifying science fields, nearly all participants were members of groups that are historically underrepresented in science fields. Most participants (75%) identified as women, and over half (51%) were first-generation college students. Participants identified as members of the following ethnic groups: Mexican American, Chicana/o, Latina/o, or Puerto Rican (32%); European American (20%); American Indian, Native Hawaiian, or First Nations (6%); Asian American (9%); African American (3%); and Multiracial (30%). In total, 70% of the undergraduate sample identified with at least one ethnic group that is historically underrepresented in science fields; the remaining 30% identified as Asian American and/or European American.

### Measures

Undergraduates participated through an online survey. As detailed below, the current research focuses on their responses to scales assessing their scientist identity and the mentoring they received. The survey they completed also assessed constructs that were not the focus of the present study such as ethnic identity, cultural values, self-efficacy, and career aspirations.

#### Scientist identity

We measured scientist identity with a scale that has been used in prior research with science-focused undergraduates (e.g., Chemers et al. 2011; Robnett et al. 2015). The prompt encouraged participants to think about their identity with the goal of “helping us understand how much you think that being a scientist is part of who you are.” Participants rated their agreement with the following six items: (1) “In general, being a scientist is an important part of my self-image,” (2) “I am a scientist,” (3) “I have a strong sense of belonging to the community of scientists,” (4) “Being a scientist is an important reflection of who I am,” (5) “I have come to think of myself as a scientist,” and (6) “I feel like I belong in the field of science.” Ratings could range from 1 (strongly disagree) to 5 (strongly agree). Internal reliability for this scale was excellent (*α* = .92).

#### Instrumental, socioemotional, and negative mentoring

We measured instrumental and socioemotional mentoring with two scales that have been used in similar studies (e.g., Chemers et al. 2011; Tenenbaum et al. 2001). We developed the negative mentoring scale for the current study. The scale development process involved consulting prior research on negative mentoring in organizational settings (Eby and Allen 2002; Eby et al. 2000) and talking with science faculty and students about common challenges in mentoring relationships.

Participants began by reading a prompt that defined *mentor* as “anyone more experienced than you who has given you individual support related to your development as a science student.” Next, they listed the names, email addresses, and university affiliations of people fitting this description. Participants who listed multiple mentors indicated which of these mentors had the most important impact on their development as a science student. Analyses focus on their ratings of the mentor they described as most important. All of the participants in the current study reported that their most important mentor was a current research mentor.

Participants used a scale ranging from 1 (definitely no) to 5 (definitely yes) to rate the extent to which their mentors engaged in various mentoring behaviors. We assured undergraduates that their responses would not be shared with their mentors. The *instrumental mentoring* scale included five mentoring behaviors such as “Helped you overcome hurdles in the research” and “Helped you improve your writing skills.” The *socioemotional mentoring* scale included 11 mentoring behaviors such as “Showed you that they cared” and “Gave you the impression they believed in you.” The *negative mentoring* scale included eight mentoring behaviors such as “Sometimes made you feel ignored” and “Sometimes made you mad.” All three mentoring scales had adequate internal reliability (instrumental: *α* = .88; socioemotional: *α* = .86; negative: *α* = .72).

## Method: mentor sample

### Mentor participants

After securing permission from undergraduates to contact their mentors, the research team sent each mentor a personalized email. The email provided a general overview of the current study. It also provided the name of the focal student who had listed her or him as an important science mentor. The email concluded by requesting that the mentor complete a brief survey that focuses on her or his relationship with the focal student. Mentors who were interested in participating used a link to access the informed consent form and online survey. As with the undergraduate participants, we assured mentors that their responses would not be shared with their students.

A total of 66 mentors participated. Most of the mentors (80%) reported that they were faculty members; the remainder described themselves as program staff (14%) and graduate/postdoctoral students (6%). Mentors’ mean age was 45.51 years (SD = 10.66, range = 29–71). Over half of the mentors (56%) identified as women. With respect to ethnic background, mentors identified as members of the following groups: European American (64%); Mexican American, Chicana/o, Latina/o, or Puerto Rican (17%); Asian American (4%); American Indian, Native Hawaiian, or First Nations (2%); African American (2%); and Multiracial (11%). In total, 37% of the mentor sample identified with at least one ethnic group that is historically underrepresented in science fields, whereas 63% identified as either Asian American or European American.

### Measures and coding

Mentors participated through an online survey. As detailed below, the current research focuses on their responses to scales assessing their mentoring behaviors as well as open-ended questions pertaining to (a) their motivation to become a mentor and (b) their views of difficulties in the mentoring relationship. The survey that mentors completed included several closed- and open-ended questions that were not the focus of the present study. These questions focused on costs and rewards of mentoring as well as strategies that mentors use to bolster their students’ self-efficacy.

#### Instrumental, socioemotional, and negative mentoring

We measured instrumental, socioemotional, and negative mentoring with the same scales that the undergraduate participants completed. More specifically, the survey prompt encouraged mentors to envision their relationship with the undergraduate who nominated them as an important mentor. With this student in mind, mentors used a scale ranging from 1 (definitely no) to 5 (definitely yes) to rate the extent to which they engaged in various mentoring behaviors. The wording for each item paralleled the items the undergraduate participants rated (see above). For instance, when rating their own instrumental and socioemotional mentoring, mentors respectively responded to items such as, “I helped this student to improve their writing skills” and “I showed this student that I cared.” Alternatively, when rating their own negative mentoring, mentors responded to items such as, “I sometimes ignored this student.” All three mentoring scales had adequate internal reliability (instrumental: *α* = .75; socioemotional: *α* = .85; negative: *α* = .73).

#### Qualitative coding

In the current study, we focus on mentors’ responses to two open-ended questions. First, we asked mentors to describe factors that encouraged them to engage in research mentoring. In analyzing responses to this question, we were especially interested in whether some mentors engaged in mentoring with the goal of furthering educational equity for members of marginalized groups. Second, we asked mentors to envision their relationship with the student who nominated them as an important mentor. We then asked them to describe difficult moments in the mentoring relationship and how these difficulties were resolved.

We used thematic analysis (Braun and Clarke 2006) to code mentors’ responses to both questions. Our approach to thematic analysis was inductive, which means that we did not have a priori expectations about the themes that would emerge. The second author began the coding process by reading participants’ responses, developing a coding manual, and coding the data. Next, the first author was trained through the three-stage process outlined by Syed and Nelson (2015); a random subset of the qualitative data was used for the training. After the training was complete, the first author and second author double-coded the remaining data (i.e., 78% of the full sample) to test for interrater reliability. We indexed interrater reliability with two methods: Cohen’s kappa (K) and percentage agreement (PA). The few disagreements (*f* = 4) were resolved through discussion and consensus.

We coded mentors’ motivation to become a mentor in two ways: (1) *equity*, in which mentors overtly described wanting to enhance the number of women or members of underrepresented cultural groups in science, and (2) *general support*, in which mentors referenced the importance of providing research resources, science opportunities, or relational support to students broadly. The motivation coding category achieved high interrater reliability (K = .91, PA = 96%).

With respect to mentors’ perceptions of difficulty in the relationship, we first coded mentors’ responses for difficulty type. Mentors reported three specific types of difficulties with their students: (1) *research underperformance*, in which the protégé exercised poor preparation, organization, or time management with respect to research tasks; (2) *communication problems*, in which mentors explicitly named ineffective communication as a problem; and (3) *personal challenges* experienced by the protégé outside of the research domain that hindered doing research. In addition, some mentors reported (4) *other idiosyncratic difficulties* or (5) *no difficulties*. Second, we coded responses for difficulty responsibility. Mentors’ attributions of why difficulties occurred, and how they should be resolved, took two forms: (1) *student centered*, in which mentors blamed students for difficulties in the relationship, and (2) *non-student centered*, in which mentors blamed themselves, larger circumstantial issues, or normalized difficulties as common to the research process. The two difficulty coding categories achieved high interrater reliability (difficulty type: K = .95, PA = 97%; difficulty responsibility: K = .86, PA = 91%).

## Results

After describing preliminary analyses, we present our findings in the following three sections. First, we compare undergraduates’ and mentors’ prevalence ratings for instrumental, socioemotional, and negative mentoring. Second, we examine the degree to which undergraduates’ and mentors’ evaluations of the three forms of mentoring predict undergraduates’ scientist identity. Third, we draw from qualitative data to provide deeper insight into mentors’ motivations to become mentors and how mentors characterized difficult moments in the mentoring relationship.

### Preliminary analyses

We began by examining bivariate correlations among scientist identity and ratings of instrumental, socioemotional, and negative mentoring. The correlation matrix and corresponding descriptive statistics are presented in Table 1. Undergraduates’ and their mentors’ ratings of instrumental mentoring were positively correlated; in contrast, undergraduate-mentor ratings of socioemotional and negative mentoring were not significantly correlated. In addition, undergraduates’ scientist identity was positively correlated with their own ratings of instrumental and socioemotional mentoring. Undergraduates’ scientist identity was not significantly correlated with their own ratings of negative mentoring; however, it was negatively correlated with their mentors’ ratings of negative mentoring.

**Table 1:** Descriptive statistics and correlation matrix for scientist identity and mentoring variables

	1.	2.	3.	4.	5.	6.	7.
1. Scientist identity	–						
2. Instrumental: student	.42***	–					
3. Instrumental: mentor	.15	.36**	–				
4. Socioemotional: student	.30*	.51***	.11	–			
5. Socioemotional: mentor	− .03	− .03	.39**	.03	–		
6. Negative: student	− .11	− .49***	.04	− .45***	.07	–	
7. Negative: mentor	− .25*	− .15	.08	− .12	− .08	.06	–
Mean	4.32	4.50	4.02	4.66	4.31	1.76	1.70
Standard deviation	.73	.71	.68	.41	.42	.56	.71
Scale range	1–5	1–5	1–5	1–5	1–5	1–5	1–5

*p < .05, **p < .01, ***p < .001

We also tested for ethnic and gender differences in the mentoring scales and scientist identity. In testing for ethnic differences, we compared undergraduates who identified with at least one *underrepresented* ethnic group (i.e., African American, American Indian; Latina/o) with undergraduates who solely identified as members of *overrepresented* ethnic groups (i.e., Asian American; European American). A main effects analysis of variance showed that scientist identity did not significantly differ on the basis of undergraduates’ ethnicity (*p* = .80) or gender (*p* = .64). Similarly, a main effects multivariate analysis of variance showed that ratings on the three mentoring scales did not significantly differ according to undergraduates’ ethnicity (*p* = .84) or gender (*p* = .88).

Last, we conducted chi-square tests of independence to examine whether the gender and ethnic composition of the mentoring dyads differed from expected counts. The chi-square assessing the gender composition of the dyads was significant. Investigation of the standardized residuals illustrated that mentoring dyads composed of a male undergraduate and a male mentor (*n* = 11; 17% of dyads) were overrepresented in the sample, *χ*^2^ (1, *N* = 66) = 5.28, *p* = .02, *V* = .28. Relatedly, there was a tendency for undergraduates and their mentors to report the same gender identity: 69% of male undergraduates worked with a male mentor, and 64% of female undergraduates worked with a female mentor. In contrast, the chi-square assessing the ethnic composition of the dyads was nonsignificant (*p* = .94): undergraduates and mentors from under- and overrepresented ethnic groups worked together in proportion to their numbers in the overall sample. However, from a descriptive standpoint, it merits noting that the majority of undergraduates from underrepresented groups (62%) worked with a mentor from an overrepresented ethnic group.

### Student-mentor comparisons

Our first research question asked whether undergraduates and their mentors perceived different levels of instrumental, socioemotional, and negative mentoring in the relationship. We assessed this question through a related-samples Wilcoxon signed-rank test. Similar in principle to a paired samples *t*-test, the Wilcoxon test allows for departures from normality in the distribution of differences between the paired variables. More specifically, for each set of paired variables, the Wilcoxon test assesses whether the difference between the two medians significantly differs from zero. Findings indicated that undergraduates and their mentors significantly differed in their ratings of instrumental (*p* < .001) and socioemotional mentoring (*p* < .001). In both instances, median ratings were higher among undergraduates than they were among mentors. In contrast, undergraduates and their mentors did not significantly differ in their ratings of negative mentoring (*p* = .26).

### Mentoring and scientist identity

Hypotheses 1a, 1b, and 1c focus on undergraduates’ and their mentors’ ratings of instrumental, socioemotional, and negative mentoring. Specifically, we predicted that undergraduates’ scientist identity would be positively associated with instrumental mentoring (H1a) and socioemotional mentoring (H1b), and negatively associated with negative mentoring (H1c). We tested our hypotheses through three multiple regressions. Regression diagnostics indicated that our data were suitable for multiple regression. Further, all variable inflation factors were less than 1.5, which indicates that multicollinearity was not a concern (e.g., Hair. et al. 1995).

In the first model, we examined whether undergraduates’ and mentors’ ratings of *instrumental mentoring* independently predicted undergraduates’ scientist identity (H1a). The model was significant, *F*(2, 63) = 6.71, *p* = .002, and accounted for 18% of the variance in scientist identity. As detailed in Table 2, undergraduates who reported receiving more instrumental mentoring tended to have higher scientist identity, which was consistent with expectations. Inconsistent with expectations, however, mentors’ ratings of their own instrumental mentoring did not explain additional variance in undergraduates’ scientist identity.

**Table 2:** Findings from multiple regression analyses predicting scientist identity from mentoring scales

	*b*	*SE*	*p*
Model 1: Instrumental mentoring			
Instrumental: student	.44	.12	.001
Instrumental: mentor	.01	.13	.98
Model 2: Socioemotional mentoring			
Socioemotional: student	.54	.22	.02
Socioemotional: mentor	− .06	.21	.76
Model 3: Negative mentoring			
Negative: student	− .13	.16	.43
Negative: mentor	− .25	.13	.05

In the second model, we examined whether undergraduates’ and mentors’ ratings of *socioemotional mentoring* independently predicted undergraduates’ scientist identity (H1b). The model was significant, *F*(2, 63) = 3.17, *p* = .04, and accounted for 9% of the variance in scientist identity. As detailed in Table 2, undergraduates who reported receiving more socioemotional mentoring tended to have higher scientist identity, which was consistent with expectations. Inconsistent with expectations, however, mentors’ ratings of their own socioemotional mentoring did not explain additional variance undergraduates’ scientist identity.

In the third model, we examined whether undergraduates’ and mentors’ ratings of *negative mentoring* predicted undergraduates’ scientist identity (H1c). Although the overall model was nonsignificant, *F*(2, 63) = 2.42, *p* = .10  (*R*^*2*^ = 7%), we did find tentative support for one of our predictions. As detailed in Table 2, mentors who reported engaging in higher levels of negative mentoring tended to have undergraduates with lower scientist identity, which was consistent with expectations. Inconsistent with expectations, however, undergraduates’ ratings of the negative mentoring they received did not explain additional variance in their scientist identity.

In sum, our hypotheses received partial support. As expected, undergraduates tended to have higher scientist identity when they reported receiving more instrumental and socioemotional mentoring; also, they tended to have lower scientist identity when their mentors reported engaging in more negative mentoring. Yet, in none of the three models did undergraduates’ and mentors’ ratings independently predict scientist identity.

### Mentor perspectives

We conclude with exploratory qualitative analyses focusing on open-ended data provided by the mentors. These findings are presented in two main sections. First, we examined mentors’ reports of why they wanted to mentor undergraduates in science (RQ2). Next, we analyzed the types of difficult experiences reported by mentors, how mentors appraised responsibility for the difficulty, and how these appraisals mapped onto their undergraduates’ scientist identity (RQ3). It is important to note that we generally did not find differences in the qualitative responses based on the gender and ethnicity of the mentors.

#### Mentor motivation

Our second research question was as follows: What motivating factors lead mentors to engage in research mentoring? We were especially interested in whether promoting educational equity was a salient motivation. Approximately one third of mentors (34.9%, *n* = 23) explained why they chose to be a mentor. To preserve a realistic account of how often each motivation occurred, the remaining percentages are reported for the whole sample (i.e., not just the sample who reported motivations). Nearly one fifth (19.7%, *n* = 13) of mentors referenced enhancing gender or ethnicity *equity* in science as the reason they became a research mentor. One male mentor from an underrepresented ethnic group explained his motivation in a way that referenced his personal experience: “It is extremely important that I facilitate the success of Native American students, as this is a way of giving back to my People, as well as repaying the debts I owe to those Native Peoples who mentored me along the way.” A more general sentiment came from a male mentor from an overrepresented ethnic group, who reported, “I wanted to help Hispanic students become scientists.” A slightly smaller group of mentors (15.2%, *n* = 10) gave *general support* accounts when explaining their motivation to become a mentor. These accounts emphasized the value of providing students with opportunities to experience and learn about science, but they did not explicitly cite equity as a motivation. For example, a female mentor from an underrepresented ethnic group expressed her desire to “make sure that our strong students are aware of the wide variety of opportunities out there, including research, study abroad, more challenging classes, so that they will be in a much stronger position when they apply for grad school and/or look for jobs.”

#### Mentor perspectives on difficulty

Our third research question focused on mentors’ accounts of difficulty in the mentoring relationship and how these accounts mapped onto their students’ scientist identity. For analyses concerning students’ scientist identity, we were especially interested in students with relatively low scientist identity compared to the overall sample mean of 4.32. Of note, students who were lower in scientist identity than their peers were not objectively low in scientist identity. Indeed, all students in the sample had sought out research mentoring, which implies at least some psychological identification with science. Accordingly, we grouped students into a *moderate scientist identity* group if they measured 4 or less on the 5-point scientist identity measure (*n* = 23, *M* = 3.48) and a *strong scientist identity* group if they measured more than 4 (*n* = 43, *M* = 4.78). As seen below, we limited these categorical analyses to comparative descriptive statistics due to small cell sizes.

The first part of our third research question was as follows: According to mentors, what are the most common sources of difficulty and conflict in the relationship with their students? Do the types of difficulties that mentors report vary depending on students’ scientist identity? Over half of the mentors (58%, *n* = 38) reported having no difficulties in their relationship. The remaining percentages are reported for the whole sample (i.e., not just the sample who reported difficulties). Among mentors who did report difficulties, *research underperformance* was the most commonly occurring problem (17%, *n* = 11). Responses focusing on research underperformance emphasized students failing to prepare, organize, or manage their research time appropriately. For example, one male mentor from an underrepresented ethnic group complained that “Garry^2^ has been and still is often unreliable. He doesn’t come in when he says he will and one time didn’t show up in lab for about three weeks.” Another difficulty involved *personal challenges* (8%, *n* = 5). For this theme, mentors described events outside of the research domain (e.g., health concerns; economic difficulties) that hindered the student’s ability to conduct research. Some mentors also described *communication problems* as a source of difficulty in the relationship (6%, *n* = 4). For instance, one female mentor from an underrepresented ethnic group noted, “I think the hardest part is my schedule is quite busy, so at certain times of the year, it can take me several days to respond to an email.” Last, some mentors (12%, *n* = 8) described idiosyncratic difficulties that were coded as “Other” due to their specificity.

With respect to students’ scientist identity, a similar percentage of mentors reported difficulties for students with moderate scientist identity (58%) and students with strong scientist identity (57%). This finding suggests that difficulty in the relationship from the mentor’s perspective does not necessarily undermine scientist identity. To understand whether conflict management could help explain mean differences in students’ scientist identity, we next coded the mentors’ appraisals of responsibility for the difficulty.

The second part of our third research question was as follows: When conflict is present, how do mentors attribute blame? Does their tendency to shoulder blame vary depending on students’ scientist identity? The following percentages are once again reported for the whole sample (i.e., not just the sample who reported difficulties). We coded mentors’ accounts of responsibility for difficulty as either student centered (12%, *n* = 8) or non-student centered (30%, *n* = 20). Student-centered responsibility occurred when mentors blamed difficulties in the relationship on the student. Oftentimes, mentors targeted the student’s personality traits. For instance, in one dyad where both the mentor and student were women from overrepresented ethnic groups, the mentor reported, “Carol tended to be ‘anal’ on some things and didn’t really want to explore. She wanted to keep using those techniques she knew well and didn’t want to learn new ones.” In addition to blaming the student for the difficulty, this appraisal style typically required the student to initiate resolving the difficulty. This was the case in one female dyad where the mentor was from an overrepresented ethnic group, and the student was from an underrepresented ethnic group. Specifically, the mentor reported, “Most problematic was when Sandra was afraid to discuss problems with me. Once she was able to talk, we were able to find good solutions.”

Non-student centered responsibility occurred when mentors attributed difficulties to things outside the control of the student. Some mentors took personal responsibility. For example, one mentor, who came from a dyad where both she and her student were women from underrepresented ethnic groups, explained, “By pointing out my own shortcomings and things that I continue to struggle with, she [the student] has been able to make adjustments in her own actions and learn to take constructive criticism rather than just becoming reactionary.” Other mentors emphasized a mutual approach to solving difficulty. For instance, in a dyad in which both parties were from underrepresented ethnic groups, the male mentor noted of his female student, “When difficult moments arise, we deal with them as if we were family, because we are a family.” It was also fairly routine for mentors to normalize difficulty as common to the practice of science, such as one male mentor from an overrepresented ethnic group, discussing his female student who was from an underrepresented ethnic group. Specifically, he wrote, “Donna, like many students, got frustrated several times while working on her research project, but we were able to talk about the fact that research is painstaking, but ultimately rewarding.”

When students were moderate in scientist identity, a similar number of mentors assigned responsibility for difficulty in a student-centered manner (17%, *n* = 4) and a non-student-centered manner (17%, *n* = 4). In contrast, when students were strong in scientist identity, mentors were three times more likely to assign responsibility for difficulty in a non-student-centered manner (35%, *n* = 15) versus a student-centered manner (12%, *n* = 5). These exploratory findings suggest that mentors might preserve their students’ scientist identity by taking a mutual approach to understanding the cause and resolution of difficulty.

## Discussion

The USA is currently struggling to populate its science workforce with qualified workers, which is a pressing economic concern (President’s Council of Advisors on Science and Technology 2012). It is critical to make better use of the available talent pool, which underscores the value of identifying ways to bolster psychological identification with science among students who are historically underrepresented in science. Findings from the current study revealed associations between scientist identity and instrumental, socioemotional, and negative mentoring in a sociodemographically diverse sample of undergraduates. The specific patterns varied depending on whether we considered undergraduates’ ratings of the mentoring they received versus mentors’ ratings of the mentoring they provided. Additional analyses provided novel insight into how mentors reason about their mentoring relationships. Below, we elaborate on these findings and discuss several key limitations.

### Are undergraduates and their mentors on the same page?

Scholars have called for additional research focusing on “matched dyadic data” from mentors and protégés (Eby and Allen 2002, p. 475). The current study responded to this call by asking undergraduates and their mentors to report on the amount of instrumental, socioemotional, and negative mentoring that mentors provided. That is, undergraduates rated their mentors along these dimensions, whereas mentors rated themselves.

Comparisons between the undergraduate data and the mentor data demonstrated that undergraduates tended to give their mentors more positive ratings than mentors gave themselves. Specifically, relative to mentors’ self-ratings, undergraduates rated their mentors as providing higher levels of instrumental and socioemotional mentoring. This pattern is interesting in light of research showing that, in workplace settings, protégé outcomes appear to be best when mentors underestimate (vs. overestimate) their own mentoring ability relative to protégé ratings (Godshalk and Sosik 2000). Other research shows that students, relative to their mentors, are sometimes more optimistic about the extent to which they mastered key skills (e.g., statistical analyses) during the research apprenticeship (Kardash 2000). The tendency for students to be more positive than their mentors may indicate that mentors are somewhat more discerning than their students. That is, when evaluating a given mentoring relationship, mentors can draw from their cumulative experiences in mentoring relationships (Hunter et al. 2007), which may allow them to make more refined assessments.

Importantly, for both undergraduates and their mentors, mean ratings of instrumental and socioemotional mentoring were at the high end of the rating scale, whereas ratings of negative mentoring were at the low end of the scale. Thus, although undergraduates provided more favorable ratings than did their mentors, both parties tended to agree that the mentoring relationships were characterized by fairly high levels of positive mentoring behaviors and fairly low levels of negative mentoring behaviors.

### Predictors of scientist identity

The current study’s primary objective was to examine whether mentoring behaviors were associated with undergraduates’ scientist identity. Our predictions originated from theory and research indicating that interactions with more experienced scientists can influence the extent to which students perceive themselves as scientists (Carlone and Johnson 2007; Chemers et al. 2011; Lave and Wenger 1991). Consistent with expectations, undergraduates who reported receiving more instrumental and socioemotional mentoring had higher levels of scientist identity. Instrumental mentoring was an especially robust predictor; together, undergraduate-mentor reports of instrumental mentoring accounted for 18% of the variance in undergraduates’ scientist identity. In this respect, findings from the current study accord with research illustrating the importance of creating opportunities for students to obtain high quality, hands-on experience with authentic research (Haeger and Fresquez 2016; Handelsman et al. 2004; Lopatto 2007).

Contrary to expectations, when undergraduates’ and their mentors’ ratings of instrumental and socioemotional mentoring were simultaneously entered into the regression models, only the undergraduates’ ratings significantly predicted scientist identity. The reverse pattern emerged for negative mentoring at a trend level: When mentors reported engaging in higher levels of negative mentoring, their undergraduates had marginally lower scientist identity. There are several possible interpretations for this finding. The possibility most consistent with our framework is that negative mentoring harms students’ scientist identity. Certainly, the behaviors captured through our negative mentoring scale (e.g., ignoring the student) could make students feel unrecognized as scientists, and may even serve to exclude them from science communities of practice (Carlone and Johnson 2007; Lave and Wenger 1991). However, we also acknowledge that we cannot rule out the reverse pathway. That is, perhaps mentors engage in negative mentoring *in response to* their students’ low scientist identity. (Correspondingly, it may also be the case that mentors who engage in high levels of instrumental and socioemotional mentoring are responding to their students’ high scientist identity.) This interpretation is consistent with social exchange theory (Cook and Rice 2003). For instance, mentors who believe that they are more psychologically engaged than their students may respond with negativity in an effort to restore equity to the relationship. A final possibility discussed below is that student-related difficulties in the mentoring relationship function as a third variable that accounts for lower scientist identity and higher levels of negative mentoring.

### Mentors’ motivation and perceptions of difficulties

Our analysis of mentors’ motivation and perceptions of difficulty in the mentoring relationship was exploratory, and small sample sizes precluded rigorous hypothesis testing. Nonetheless, the qualitative findings align with prior research and reveal important suggestions for future research. Over half of the mentors who responded to the question about their motivation to become a research mentor expressed a desire to enhance gender or ethnic equity in the sciences, whereas the remaining accounts focused on more generally supporting students’ access to scientific training. Across the qualitative data, we found few differences based on the gender and ethnicity of the participants. For example, mentors from underrepresented backgrounds did not appear to endorse equity as a reason for being a mentor in higher numbers than mentors who were from overrepresented groups. Nevertheless, some individual accounts suggested that mentors from underrepresented groups pursued equity from a more personal standpoint, whereas mentors from overrepresented groups pursued equity as a more abstract, ideological goal.

Mentors’ accounts of difficulty in the mentoring relationship most commonly referenced problems with research underperformance, followed by personal challenges that were external to doing research. These findings, which came from mentors reflecting on a single mentoring relationship, mirror findings that Eby and McManus (2004) obtained when they asked workplace mentors to reflect on negative experiences across all of their prior mentoring relationships. Several mentors in the present study also described communication problems with the focal student as a key source of conflict in the relationship. This is noteworthy in light of research showing that undergraduates who feel unsupported by their mentors often cite miscommunication as a cause of conflict (Haeger and Fresquez 2016).

Mentoring challenges were present regardless of whether undergraduates were moderate or strong in scientist identity. However, when undergraduates were strong in scientist identity, their mentors appeared to be more likely to appraise and resolve the conflict with a non-student centered approach. That is, mentors took personal responsibility for the negativity, took a collaborative approach to resolving conflict, or located the difficulty in some circumstance external to the mentoring dyad. A non-student-centered approach conceivably builds trust and closeness with students, which may help to preserve students’ scientist identity in the presence of conflict.

This possibility aligns with work showing that conflict is not necessarily a problem in itself (De Dreu 2006; Simons and Peterson 2000). Indeed, conflict may contribute to positive outcomes when it is accompanied by conflict management that is agreeable, collaborative, and accommodating (see DeChurch and Marks 2001). Importantly, however, conflict management strategies appear to vary cross-culturally (e.g., Leung 1987) and on the basis of gender (e.g., Holt and DeVore 2005). This may contribute to miscommunication between students and mentors who do not share the same background (see Haeger and Fresquez 2016). These misunderstandings could be especially detrimental to students who are historically underrepresented in science. Accordingly, we echo Haeger and Fresquez (2016) in noting that it would be beneficial to offer mentors training in culturally sensitive communication and conflict resolution. The current study’s findings indicate that at least some mentors would be open to this type of training, given that a sizeable minority reported that the goal of fostering educational equity drives them to engage in research mentoring.

### Methodological recommendations

On the basis of the current study’s findings, we provide several methodological recommendations. First, prior research indicates that there are advantages to collecting data from both protégés and mentors (Eby et al. 2010; Hunter et al. 2007; Kardash 2000). Findings from the current study yielded mixed support for this premise. On the one hand, undergraduates’ and mentors’ ratings did not independently predict scientist identity. That is, when included in the same regression model, undergraduates’ ratings and mentors’ ratings were never both significant predictors. On the other hand, however, scientist identity was associated with *undergraduates’* ratings of instrumental and socioemotional mentoring, but *mentors’* ratings of negative mentoring. Moreover, mentors’ open-ended accounts provided meaningful contextual information that could not have been obtained from undergraduates. Therefore, collecting data from just one person in the mentoring relationship would have yielded some, but not all, of the findings we obtained in the current study. Given that collecting data from student-mentor dyads is labor-intensive, we encourage researchers to be especially strategic about the questions they ask students and their mentors to ensure that they utilize both sources of data to their full potential.

Our second set of recommendations pertains to negative mentoring. We echo others in noting that negative aspects of mentoring relationships merit additional attention in future research (Eby et al. 2010; Scandura 1998). Given that the overall model for negative mentoring was nonsignificant in the current study, an important first step for future research is to refine our negative mentoring scale with the goal of capturing a wider array of negative mentoring behaviors. Further, we encourage researchers to consider both mentors and their students as potential sources of conflict in the relationship. Whereas our quantitative measure focused on negative behaviors on the part of the mentor, qualitative data from mentors suggested that students can also engage in behaviors that undermine the mentoring relationship (see also Eby et al. 2010). For instance, some of the mentors in the current study reported that underprepared students were a source of conflict in the relationship. In future research, it would be worthwhile to assess the dynamic interplay between negative student behaviors (e.g., being underprepared) and negative mentoring behaviors (e.g., ignoring the student). It also merits noting that conflict is a normal part of interpersonal relationships (Wood and Duck 1995). Thus, it seems plausible that student outcomes are not influenced by challenges in the mentoring relationship per se; rather, student outcomes may instead be influenced by the mentor’s approach to managing conflict (e.g., constructive and culturally sensitive vs. critical and culturally insensitive).

### Limitations and future directions

Findings from the current study need to be considered alongside several key limitations. First, our sample size was fairly small, which reduced statistical power. Further, the small sample size precluded analyses that assess whether scientist identity and mentoring behaviors vary as a function of the sociodemographic breakdown within the mentoring relationship (for discussion, see Blake-Beard et al. 2011; Ensher and Murphy 1997). For instance, it would be interesting to know whether the association between mentoring and scientist identity varies in strength depending on whether the mentor and student share the same ethnic or gender identity. Relatedly, findings from the current study revealed a tendency—particularly among men—for students and their mentors to have the same gender identity. Future research should attempt to explain why this is the case and examine whether mentoring practices differ based on the gender composition of the dyad.

Second, it is important to be cautious about generalizability. We recruited undergraduates from a research conference, and most of our participants presented at this conference. This suggests that our participants were not only more motivated than the average undergraduate, but also more successful researchers than many students who work in research labs. Relatedly, undergraduates tended to rate their mentors very favorably. It is plausible that undergraduates who had less positive mentoring experiences either self-selected out of the study or did not feel comfortable allowing the research team to contact their mentors. The low base rate for negative mentoring may help to explain why undergraduates’ ratings of negative mentoring were not significantly associated with their scientist identity. In future research, it would be worthwhile to collect data from students with less favorable mentoring experiences. We suspect that findings would indicate that negative experiences with mentors can seriously undermine undergraduates’ burgeoning scientist identity.

Another limitation pertains to our focus on undergraduate-mentor dyads. In a recent study, Aikens et al. (2017) observed that research mentoring relationships are often triadic, such that they involve faculty, graduate students, and undergraduates. In *open triads*, graduate students serve as an intermediary between faculty and undergraduates. Conversely, in *closed triads*, undergraduates interact with graduate students and faculty. Closed triads appear to have especially positive implications for scientist identity. Future research should examine whether different types of mentoring have different implications depending on who in the triad provides them.

A final limitation is our reliance on a self-report scale to measure scientist identity. Although this is a common approach (e.g., Chemers et al. 2011; Hazari et al. 2010), our conclusions would have been strengthened through the inclusion of objective outcome variables that covary with scientist identity. For instance, among the juniors and seniors who participated, it would have been interesting to examine whether mentoring experiences are associated with their number of applications and acceptances to graduate school (see Estrada et al. 2011). More generally, following students longitudinally to examine the extent to which different forms of mentoring (e.g., instrumental vs. socioemotional) predict retention in the science pipeline would also be a worthwhile direction for future research.

## Conclusions

The current study suggests that instrumental, socioemotional, and negative mentoring behaviors may have implications for scientist identity among undergraduates who are historically underrepresented in science. Of the three forms of mentoring, instrumental mentoring explained the most variance in undergraduates’ scientist identity, which suggests that it is especially important for undergraduates to receive skill-based guidance from their mentors. The associations between mentoring and scientist identity varied depending on whether we considered mentoring evaluations from undergraduates versus their faculty mentors. Moreover, qualitative data from mentors revealed challenges in mentoring relationships that were not captured through the quantitative data from undergraduates. Thus, from a methodological standpoint, future research may benefit from taking an approach that can provide insight into the dynamic complexity of mentoring relationships.

## Footnotes

1 The outreach organization explicitly caters to students who identify as Hispanic, Chicana/o, and Native American, but it includes students from a wide variety of ethnic and cultural backgrounds.

2 All names have been changed.
